# Women coaches leadership development programme: an evaluation study of programme effectiveness

**DOI:** 10.3389/fspor.2024.1433787

**Published:** 2024-09-23

**Authors:** Sophia Jowett, Katelynn Slade, Jyoti Gosai, Louise Davis

**Affiliations:** ^1^ School of Sport, Exercise and Health Sciences, Loughborough University, Loughborough, United Kingdom; ^2^ Department of Psychology, Nottingham Trent University, Nottingham, United Kingdom; ^3^ Department of Psychology and Umeå School of Sport Science, Umeå University, Umea, Sweden; ^4^ Department of Psychology, Swansea University, Swansea, United Kingdom

**Keywords:** women, leadership, gender, coach, workforce, evaluation

## Abstract

**Introduction:**

Women's sport has been experiencing continuous growth, yet the low levels of women coaches in the United Kingdom (UK) suggests that the sport is missing out on potential talent. Guided by empirical research, a women-only leadership development programme was designed and implemented by UK Sport to a cohort of 20 coaches from January to June 2021. The main characteristics of the programme included raising awareness of gender bias while at the same time focusing on women coaches' leadership purpose and skills within a safe environment that supports coaches to build their leader identity.

**Methods:**

Kirkpatrick's (1998) four-level model was employed to guide the evaluation of programme effectiveness: reactions, learnings, applications and results. Interviews were conducted with 17 participating coaches.

**Results:**

Content analysis of the qualitative data revealed five general categories (a) evaluations were mainly positive highlighting the aspects of the programme they liked, enjoyed and made most impression onto them; (b) learnings that impacted their work directly and immediately emerged as was the importance of on-going development; and (c) the majority of the women desired progression and transitioning to senior coach leadership positions was felt within their grasp; nonetheless, (d) challenges emerged and were described as organisational (e.g., recruitment, remuneration) and personal (e.g., work-life balance, childcare).

**Discussion:**

Overall, the effectiveness of the programme was captured in its capacity to raise awareness, develop knowledge, build connections, and inspire so much so that its effects translated to many of these women moving to more senior leadership positions post-programme. Practically, this evaluation highlights that investment in individual coaches is part of the systemic change required to bring about gender balance in the coach workforce.

## Introduction

Women's sport has been experiencing continuous growth. The participation of female athletes has increased so much that Paris 2024 is expected to achieve full gender parity for the first time in the Olympic Games history (1). Nonetheless, the number of female coaches has remained extremely low in most parts of the world. There are numerous reports including the IOC Gender Equality Review (2) and the UK Coaching Coach Workforce (3) that evidence the degree to which women are underrepresented in coaching especially in performance contexts [see also (4)]. In fact, researchers have highlighted that the numbers of women coaching are in decline or at best in a stagnant trend [e.g., (5–7)]. A fundamental question is, “Why are women so poorly represented in high-performance sport coaching?”. Over the years, numerous explanations have been put forward for the underrepresentation of women in coaching; these include low levels of intention to coach, motivation and even a lack of competence and confidence has been mentioned [e.g., (8)], among other reasons such as organisational and cultural [e.g., (9)]. Moreover, it has been suggested that women coaches face fewer coaching opportunities and exposure, unequal performance standards, poor working conditions, lack of connectivity and recognition as well as hostility, isolation, exclusion, devaluation, rejection, and sexism [e.g., (10–13)].

Research findings suggest that diversity and inclusion in sport, as in other domains of professional life [engineering, medicine, armed forces; e.g., (14)], can increase the talent pool, improve service, enhance image, promote creativity and problem-solving, improve decision making, enhance relationships, satisfaction and commitment within the workforce (9, 13, 15). Subsequently, striving for a gender-balanced coach workforce makes both business and moral sense that can lead to healthier and better performing workplaces. In the UK, several sport organisations (e.g., UK Sport, Sport England, UK Coaching) have highlighted their intentions for a more inclusive and diverse workforce. For example, in the UK Sport Strategic Plan 2021-31 (16), the mission is defined by three ambitions (i.e., winning well, thriving sporting system, inspiring positive change) and within each of these ambitions the common thread is nurturing collaboration, connection, inclusion, diversity, positive influence, and ethical environments whereby each and every-one person within the sport system thrives. With this strategy in mind, UK Sport felt that it was more important than ever before to tackle shortages in the coach workforce especially within the context of high performance sport (17). UK Sport made a public promise in 2021 to double the number of women coaches in the Olympic and Paralympic Games in Paris and to ensure that more women coach in talent pathways by 2024.

To fulfil this promise, UK Sport consulted academic researchers to design an evidence-based women-only coach leadership development programme. This leadership programme formed part of a series of other initiatives led by UK Sport, alongside Sport England and other home nations (Scotland, Wales, and Northern Ireland), as well as UK Coaching and Chartered Institute for the Management of Sport and Physical activity (CIMPSA: UK's professional body for the sport and physical activity sector). Collectively, these initiatives have run for over four years (2020–2024) and have aimed to increase the number of women coaches, provide continuous professional development, raise awareness about gender bias, emphasize the importance of inclusivity, and ensure representation at all levels and types of sport. While these initiatives have provided additional support to women coaches within the UK sport system, they have also provided an opportunity for everyone involved in sport to consider ways to support women coaches to evolve within their respective organisations.

In this article, we will focus on one of the initiative that was put in place with the objective to fill the pipeline and produce parity up through the ranks. This initiative involved the development and implementation of a leadership development programme for women coaches, known as the *Female Coaches and High-Performance Leadership Programme*. This leadership development programme was targeted at women who were believed to be ambitious to coach at the highest level of performance and considered by their respective line managers and/or senior colleagues within their national sport organisations approximately three to four years away from coaching at Olympic and Paralympic Games. The development programme ran for the first time in January 2021 and was repeated in January 2022 by UK Sport. Each time the programme was implemented, it was delivered for a total duration of six months with a cohort of 20 and 24 coaches, respectively. The programme was developed and delivered by an academic (first author) in association with a mentor and seven master coaches. The mentor was a retired performance coach whose work revolved around supporting and mentoring leaders in and outside of sport, while the master coaches were active performance coaches in Olympic or Paralympic programmes. In this paper, we focus on the evaluation that took place at the end of the first implementation of the programme in January 2021. (Note. The programme ran in line with the UK government regulations during the COVID-19 pandemic).

The *Female Coaches and High-Performance Leadership programme* was not based on a framework that adopts an “add-women-and-stir” approach whereby the emphasis is on delivering the same programme to women that was intended to be delivered to men. Such an approach suggests that gender does not or should not matter for leadership development. Moreover, this programme was not based on a framework that adopted a “fix-the-women approach” whereby the emphasis is on gender matters a great deal, but the problem is placed on women (18). This approach is based on a rather out of date idea that suggests that women have not been socialised to compete successfully in the world of men and so they need to acquire skills men have to compete against them [see (19) for an excellent treatise]. While both these approaches have aspects that can be useful to women (e.g., decision-making, feedback, support), they do not focus on the organisational realities women face that would be helpful to their leadership as coaches in the long run [see e.g., (20–22)]. Instead, the framework used to guide the design of UK Sport's *Female Coach in High-Performance Leadership Programme* was based on specific leadership factors that women need to leverage to be effective as head or assistant coaches within their national sport organizations, where the focus is on high performance.

Subsequently, the programme zoomed in on identity and gender bias to highlight how the latter can interfere with identity-building while honing on three sets of leadership skills: (1) vision, (2) networking and (3) negotiation. Vision including self-identity [see (23–25)], networking [e.g., (26–28)] and negotiation [e.g., (29, 30)] were key topics that were deliberately chosen. These topics were employed because women coaches do not naturally think of these skills as important to them. Moreover, these topics were reinterpreted through the lens of stereotypical bias and specifically second-generation bias. According to Ely and colleagues (2011), “second-generation” forms of gender bias refer to powerful but *subtle* and often *invisible* barriers that arise from cultural assumptions and organisational practices and patterns of interaction that unintentionally benefit men while putting women at a disadvantage. Thus, the reinterpretation of vision and identity as well as networking and negotiation through the lens of such gender biases aimed to (a) promote women coaches’ identity work and (b) provide an alternative perspective of how such leadership skills can be used to assist movement into more senior coach-related and leadership roles.

The *Female Coaches and High-Performance Leadership programme* lasted six months in duration and included three by three-hour interactive lecture/seminar sessions, personal reflections, assignments, work shadowing and small group sessions. The group sessions were facilitated by the mentor who was a high-profile former female Olympic coach with experience in executive coaching. The small group sessions were an integral part of the programme and aimed to help participating coaches to engage in sense making of the main topics discussed in class (i.e., identity and stereotypes, vision, networking and negotiating) while work shadowing supplied additional opportunities to explore the environment of high performance at a more local level. Work shadowing was facilitated by the master coaches who at the time of the implementation of the programme were Olympic and Para-Olympic coaches in leadership (head coach) positions in sports such as athletics, gymnastics, judo, diving and swimming as well as para-triathlon and boccia. According to Simkins and colleagues (2009), work shadowing allows opportunities to explore and understand transition and capability, as well as identity. The participating coaches also had the opportunity to present their six-month learning journey and capture their before, during and after reflections to an audience comprising of their mentor, master coaches, and their sponsors or line managers (i.e., senior members of staff within their respective national sport organisations) who put them forward for the leadership programme in question.

When the first *Female Coaches and High-Performance Leadership programme* completed in 2021, the women coaches who formed the cohort of 20 participants were asked to evaluate the programme. Most of the participants (85%) took part in semi-structured interviews with the following aims: (a) to evaluate the programme in terms of its effectiveness; (b) to explore their current professional standing and ambitions in terms of how these evolved over the six months, as well as (c) to consider the support they may require in terms of achieving their professional goals beyond the programme. These interviews were designed to capture participating coaches’ appraisal regarding what worked and what did not work for them as individuals and as members of the first cohort of this programme. In 2024, they were contacted again to obtain information about their career progression. The significance of this evaluation lies in refocusing activity on aspects that can support women coaches as leaders by creating a basis for future planning that is both effective and impactful. There is scope for research that evaluates the efficacy of coach development programmes, and the long-term impact women-only leadership development programmes have on coaches (31, 32).

## Methods

The aim of the research was to determine the effectiveness of the *Female Coaches and High-Performance Leadership programme*. It was posited that the coaches’ perceptions (evaluations) were a result of the experience of the programme. A qualitative approach was therefore needed to encourage participating coaches to describe their experiences and an interpretative approach was also needed to analyse their descriptions and perceptions. It was deemed central that impactful learning and development was dependent on the relationship established between the coach (experiencer) and the programme (experienced) [cf. (33)]. This ontological stance supports an epistemological stance of learning and development through subjective interpretative sense making and meaning that allows coaches to recall and describe their experiences. Moreover, qualitative content analysis was employed as an interpretive approach to highlight that interpretations are co-creations of the interviewee (coach) and the interviewer (researcher). Thus, the interpretations during the analysis phase is a co-creation of the researchers and the obtained transcripts or texts (34).

### Participants

From the 20 coaches that took part in the leadership programme^1^, 17 (85%) participated in this evaluation study, yielding a high response rate (35). Participants’ ages ranged from 25 to 58 years (M = 37.63; SD = 9.85) and represented athletics, alpine skiing, canoeing, cycling, diving, fencing, goalball, golf, hockey, rowing, and swimming. Participants were working in a range of coach-related roles including talent lead, performance coach, pathway coach, personal/individual coach, academy coach, analyst lead and coach, assistant coach, athlete development programme coach, talent identification (ID) coach for a World class Programme, national coach, and speed coach. The qualifications and experience of coaching varied with the majority possessing a university degree (not necessarily sport-related degree) and /or coach-specific certification (e.g., Level 2, 3) and coaching from three to 20 years (M = 10; SD = 2.55). To ensure confidentiality of the participants’ responses, codes are used to refer to the participating coaches (the capital C to denote Coach and numbers to refer to the different coaches).

### Instrumentation

Kirkpatrick’s (36) four-level model was employed to guide the evaluation of the programme effectiveness: (1) reactions—what women coaches thought and felt about the leadership development programme; (2) learning—the resulting increase in knowledge or capability; (3) improvement and application of knowledge/skills acquired; (4) results—the effects on the environment or organisation resulting from the women coaches’ knowledge and learnings including their overall performance. Subsequently, the semi-structured interview schedule developed consisted of four open-ended questions reflecting the four levels with additional prompts and probes as deemed necessary during the interview. The questions were developed based on Kirkpatrick’s (36) four-level model and aimed to gather relevant information concerning the effectiveness of the programme. The main questions revolved around asking participants about:

• what were your general impressions of the programme (e.g., likes and dislikes, what could have been done better or more of);
• what have you learned and what has made the greatest impression on you during the programme;
• how the knowledge and insights gained from the programme would contribute to fulfilling your personal ambitions after the programme;
• what recommendations do you have for improving the quality of the programme and what should support look like beyond the programme.

In 2024, all 20 coaches were contacted again via email asking them two questions: (1) Are you currently working (as a coach)? and (2) Is your position/role within your organisation today more senior than the role you had when you participated in the leadership development programme between January and June 2021? These questions aimed to capture the potential long-term impact of the programme on their personal performance and on their sport organisation.

### Procedure and data analysis

After gaining ethical approval from Loughborough University, participants were interviewed virtually (via Microsoft Teams) by the first author with interviews lasting between 48 and 71 min. Subsequently, all interviews were transcribed by the second author, generating 290 double-spaced pages of transcripts. Qualitative content analysis where data are represented in words and themes was employed as the main analytical method [see (37)]. Guided by the work of methodology scholars (38–43), the following phases were employed to content analyse the obtained qualitative data: (a) familiarisation with the data by reading through the transcribed text and obtaining an overall picture of “what is going on?”; (b) identification of relevant common patterns, themes, and categories guided by the four main questions from the interview schedule (i.e., reactions and first impressions of the programme; main learnings and knowledge gained; application of acquired knowledge and skills); this stage of the analysis generate five general categories: *reactions*, *learnings*, *ambitions*, *challenges* and *recommendations*; and (c) further classification of general themes and categories to more specific categories, namely sub-categories or sub-themes. In addition, the information gathered about coaches’ current professional roles within their designated sport—nearly 3 years on from the time the programme was delivered to potentially capture tangible impact [cf. (44)] formed one single category.

A number of strategies were employed to maintain trustworthiness (45–47): (1) there was “prolonged engagement” between the first author and the participants, rapport was existent at the time of the interviews, though the author remained cautious to not become so immersed that their professional judgement was influenced; (2) honesty and openness from the participants were sought—each participant was given the opportunity to participate voluntarily and to refuse participation unless they wished to offer their data willingly and freely; (3) member checks took place during the interview and occasions to discuss the data were presented with the participating coaches post interview to ensure the accuracy of the findings—these actions did not influence the data analysis process; and (4) opportunities for scrutiny of the project by colleagues and peers were embedded providing opportunities for reflection, challenging assumptions, and strengthening the rigour of the research at different phases of the project. The results of the analysis are discussed next.

## Results

This section revolves around the four main questions or areas the interview targeted: reactions to programme, recommendations, ambitions, and further support. Before the specific findings of the targeted areas are closely examined, it is important to highlight that all participants assessed positively the *Female Coaches High-Performance Leadership Development programme*. For example, C3 showed her appreciation as follows,

“I just suppose that a huge thank you…thanks very much for having me on the programme and also thank you for all your hard work and your teaching and organising all the groups and getting everything set up. I mean, it is this it’s been great for me. I've really learnt a lot. So, thank you very much for the opportunity and yeah, really looking forward to keep in contact with everybody and, you know, all the other women that we've made connections with on the WhatsApp groups and things like that. So, yes, it’s been a really nice community around that. So, I'm really looking forward to carrying on”

Other participants, such as C6 expressed similar sentiments by saying, “it's been really good for me to be part of it… I've definitely learned a lot. And if there's any more opportunities I’d really like to continue moving forward.” and C10 asked, “Please, can we do some more?”, while C12 rated the programme, “Ten out of 10”.

The deductive analysis of the obtained qualitative data led to the creation of five main categories: (1) *reactions* (to the programme) that contained “like” and “dislike” elements of the programme; (2) *learnings* from the programme that revolved around areas discussed and knowledge gained that were readily applicable into the environment or place of work; (3) *ambitions* that focused on professional goals and plans for achieving them; (4) *challenges* comprised of organisational and personal aspects needing careful consideration and navigation; and finally (5) *recommendations* related to what can be done to ensure coaches find themselves in positive networks and continuously develop their knowledge and skills among others. These categories are presented below.

### Reactions

Participants reacted to the programme by expressing “likes” and “dislikes”. Participants identified four key areas that were *liked* or favoured about the development programme: the three separate strands of the programme (i.e., theory sessions, small group and work shadowing sessions), sharing and learning from each other, the friendly and social environment created, and the people on the programme (see Table 1). For example, one of the strands of the programme was mentoring and the sentiment was that both the structure and delivery were of a very high standard; a coach said, “[the mentor] was great; you just can't get away with anything with [the mentor]… I think it was easy to be vulnerable with her and at the same not feel that you’ve been vulnerable.” C16, and another said, “I felt that these sessions were quite thought-provoking…” C2; and last but not least, “the mentoring sessions pushed me, I suppose, a little bit out of my comfort zone… kind of forced me to reflect… and be open to feedback” C14.

**Table 1:** Summary of the content analysis: reaction (to the programme).

General category	1st order	2nd order
Reaction	“Like”	3 strands (theory, master coaches, mentor sessions)
		Sharing, learning, and networking with cohort (including one-to-one discussions, WhatsApp group chat)
		Environment (small group/breakout sessions, safe environment, virtual and easily accessible)
		Cohort (Women only, diversity of sports represented)
	“Dislike”	Cohort (lack of engagement/ connection in person, disparity between experience)
		Arrangements with master coaches
		WhatsApp group chat (dominated by some)
		Lack of face to face
		Length of programme; too short
		Lack of interconnectedness to the wider group

The theory sessions were liked for the opportunity to digest new information and insights, as well as consider alternative perspectives (e.g., their broader role as a leader within their organisation) while providing the space to personally contemplate and reflect. For example, C3 said, “I enjoyed listening and learning and reading about things and reflecting on my own is probably something that I quite enjoyed doing”. Participants expressed their gratitude for exposing themselves to information never explored before around vision and identity, as well as networking and negotiation. C11 explained, that the theory sessions were, “brilliant and really thought provoking for me.” While C17 highlighted “I like the topics, they contained useful practical insights around negotiation and networking. I used to think I loathed negotiating…and then the programme made me realise how good I am at networking and in fact I love it”. Moreover, C7 mentioned that the three strands of the programme were felt to be connected well together,

“…these three strands [theory, mentoring, master coach sessions], the way they interacted and didn't just leave a subject sort of talked about, discussed and then left on the shelf, I think it was brought into life. And I think it's quite easy to feel like you've covered something by doing several hours sort of it. But actually, it was the smaller groups and the tasks set following the theory sessions for example, but especially the smaller groups that kick started that reflective practise or reflective thinking, on the topics delivered”

The master coach work shadowing sessions were well-received by most of the participants. For example, C14 stated, “I work best in one-to-one situations and smaller groups, I like personal conversations.” and C2 highlighted “I think she [master coach] added a huge amount of value for me and also made me realise that I was good at what I do… I think she made me realise that I was good and that with or without them, I’m still going to be good.” And C3 expressed the impact of her master coach on her personally “even just half an hour with [master coach] and she's giving me so much useful information, I'm looking back and I'm thinking why did I never think about that… for example she advised me identifying a coach developer, which is something that I haven't thought about.”

Participants also highlighted the environment and the cohort, the people therein, as being positive aspects of the programme. For example, C15 talked about the small groups formulated within it, “having a small group, you felt you got to know people at a personal level. And then that meant that when you were reflecting, you felt safe and had a personal connection” and “So you've created a really safe environment where these women can share their vulnerabilities… I think here you could be yourself, share your vulnerabilities.” Participants expressed the sentiment of wanting the programme to be face to face but C4 highlighted the added benefit of the virtual sessions, the added flexibility and not having to choose between training sessions and personal development or having to think the logistics of travelling and so on, “what I thought was really good was that it was online… I could just log on at home, and it was there and I found that great.” C6 also added “just on the online stuff, I actually think that people think quite negatively about connecting online… But to get that amount of people in one place at the same time is complex; it is actually more efficient”.

Lastly, the participants cited the cohort and sharing, learning, and connecting with individuals within the group as another positive aspect of the programme. For instance, C5 said, “the fact that it was an all-female course is really valuable” and “now for me, I've got contacts with, you know, gymnastics and I'll say trampolining… for me, it's just been one of those where it's more connections [and] ultimately that's what makes this world work and that's what makes you get to the next spot sometimes.”. The diversity of sports represented in the cohort was considered favourably (e.g., “And the fact that it was all different sports, I think was really brilliant.” C10). It was also highlighted that learning, exchanging information and connecting with the other members of the programme was facilitated via the WhatsApp group chat. For example, coach C13 stated that a lot more content was shared in the WhatsApp group chat, “I would never have probably seen or read, and they were really thought provoking and inspiring.” C15 talked about the added benefit of learning from others on the programme, “But what I found useful was listening to everybody and then getting fresh insights and making it relevant to my journey” with C2 sharing that same sentiment, “I like to listen to how other people worked out the journeys that they were going on as well”.

Participants highlighted dislikes or aspects of the program that were least favoured. For example, some individuals in the cohort raised concerns about the mode or type of engagement, finding it ineffective or irritating. Specifically, in the WhatsApp group chat “some of it was annoying when people kept putting stuff in there” C11. In terms of the all-female programme one participant said, “I’m not used to just women. And obviously there's some big characters that are like much more vocal and throwing stuff in all the time.” C8. On a different yet related note, a coach (C17) also felt it important to clarify that the purpose of the *Female Coach Leadership Development* program is not to promote women regardless of their competence, rather “it aims to empower women to achieve their fullest potential, ensuring they don't feel like an add-on or an out-group, especially when most of sport consists of male coaches. Instead, they should feel very much a part of it.”

Another participant highlighted the lack of face to face, as well as the length of the programme (e.g., “I thought it was just too short.” C11). Regarding the engagement and connection with some of the cohort members, participants stated, “there were quite a few that were cagey, didn't say anything” C1 and, “I struggled in my group because the other girls didn't really say anything… I spoke to the mentor about it” C11. One participant talked about the range of experience between the cohort members as something she struggled with during the breakout groups, “because I feel like I don't get anything out [of it]. I feel like I’m bit of a mentor for some of these women, which is fine”.

Although the master coach sessions were highly rated by the participants, there were some aspects that the coaches of the cohort found ineffective, including either the timing or lack of time of the master coaches [because of the upcoming Olympic and Paralympic Games in Tokyo 2020 (summer 2021), master coaches were understandably too busy, C8, C9, C11], lack of face-to-face, and not connecting personally with some master coaches. C8 and C13 lacked connection and deeper learning with their master coaches, whereby C8 explained,

“because I hadn't seen her work, I couldn't pick out what I thought she was really good at or ask her questions about that. I think in hindsight, if I had been able to go and watch her at work, I would have been far better at questioning her and having great questions for her than I was…I didn't feel like I knew enough about her and what she did and how she did it”.

Also, C13 stated that “[I] expected a bit more” with the master coach sessions and wanted the master coach “to dig a bit deeper” and go beyond the “the surface of it” C13. The virtual master coach element in her case prevented exploring areas of interest in sufficient depth and breadth. Finally, C17 highlight that more than 2–3 coaches are assigned to master coaches so that a community/group is formed (e.g., “if one drops out you are on your own whereas with a bigger group if one drops out, you are still a group of coaches attending the master coach sessions”).

### Learnings

During their interviews, participants had the opportunity to discuss what they learned from the program and how they applied it in their environment or place of work. Reflecting on what they learned, participants main takeaways were centred around self-reflection and interpersonal effectiveness (see Table 2) including having a vision, authenticity, building their identity, increased confidence, and self-belief, discovering own path (and specific steps needed to follow it), prioritising self-development (e.g., “I did put time in the diary for things like reflecting.” C5) and reaffirmed their thinking and broader values that defined who they are and why they do what they do. A key learning for C3 from the programme was that she can be her “authentic self”, “I can be more just myself and just be a little bit more strategic with the way I apply myself… I don't necessarily need to be pushy and overbearing, and the kind of characteristics that I feel males just get away with”. While C8 explained how the programme helped her realise that she had “pressed pause” on her vision due to family and childcare needs, however, the programme prompted her, “just go and do everything you can to be the best you can be”. It also helped to remind her, that although athlete care is important, her self-care and self-development is important too, “So I think it just hammered at home with me that I become very focussed on athletes and not so much my own development… reminded me how to develop and how to learn”.

**Table 2:** Summary of content analysis: learnings and takeaways from the programme.

General category	1st order	2nd order
Learnings/takeaways from programme	Self-reflection and self-improvement	Importance of self-reflection
		Increased self-confidence
		Finding self-belief
		Authenticity
		Importance of having a vision, a purpose
		Discovering own path and specific steps needed to take to achieve it
		Prioritising self-development
		Reaffirmed thinking and values
	Interpersonal Skills	Recognising and appreciating others’ perceptions
		Importance of wording in conversations
		Power of networking
		How to negotiate
		Different ways to interact with their athletes
		Being clear when communicating with others

The leadership skills learned or developed by the participants during the program had a lasting impression on them. Moreover, the realisation of how second-generation bias or subtle barriers can personally and profoundly affect them including their identity formation was revelatory. The discussions around the paucity of role models for women, gendered career paths, lack of access to networks, sponsors, and double binds expanded their insights into themselves and their organisations. In effect, the skills and insights enabled them to plan their career journey more effectively. For example, the importance of developing and articulating a vision, building a network, and understanding how to leverage it, using negotiation skills to communicate clearly and convincingly for a position, or for physical and human resources offered opportunities to consider and practice these skills in their environments. For example, C16 realised for the first time that “your own perspective is not necessarily what other people are experiencing… just made me really question decisions I have made in the past.” and “When you network, you must give.”, while C6 said, “I kind of understand what I need to do, I need to be really clear about what I want professionally and I need to network more.”

### Ambitions

Participants were asked about their ambitions. The focus here was to establish how these participating coaches were thinking about the future at that present time. They were also asked to consider, where they would like to be in three to four years’ time. Four sub-themes emerged from the analysis: importance of ongoing self-development, capacity to influence sport, progressing (transitioning) in the job role, and gaining added-value experiences such as attending to and participating in major competitions (see Table 3). Participants highlighted the need to focus on on-going self-development as a personal and professional goal. Development was viewed as a means to further build their self-confidence and belief (e.g., “My aim is [to]…go into Beijing in a more confident position as a coach” C9). They also referred to advancing and progressing beyond their current role and higher up roles as an ambition, “I see myself being in the role that I’m in now…I’m quite keen to build on the learning and experience and grow my understanding of what my programme and coaching looks like.” (C7). Moreover, participants expressed a desire to influence their sport by making history. They aim to become more well-known as women coaches, leaving behind a legacy, and becoming role models for others to follow. They also aspire to move into roles never before achieved by a woman, all while contributing in a positive and significant way to the growth of their own sport. One of the coaches said, “I want to make history and be the first female coach to stand on the European [stage]…being female coach to a male player.” (C12) and C9 talked about developing the sport she is involved in so that there are “more athletes involved in it….[by] making a clear development pathway through from the clubs and the academies up into the World Class Programme.”

**Table 3:** Ambitions and action plan.

General category	1st order	2nd order
Ambitions	Self-development	Increase self-confidence
		Develop self within current role
	Influence sport	Make a difference to the sport in terms of diversity
		Influence positively the mission of the sport/ organisation
		Impact one's own sport (working with athletes)
	Consider current and future Job Roles	Aspire to a senior role (High performance coach, Head coach, Performance director, National coach, Senior/international/ podium level coach, Lifestyle role
		Develop in a current role
	Attend a major competition (at junior or senior level)	Gain experience within HP (Olympics/Paralympics, European Championships, World Championships)
Plan of action	Knowledge building	Attending courses (such as HIPAC); Shadowing those already in role; Having a coach developer; Learning about aspired role; Attending conferences; Knowing what HP looks like; Increasing tactical/technical knowledge; Reading papers
	Experience	Gain leadership experience; Attend a major competition; Change environment (sport/country); Gaining experience in the aspired role when given the chance; Demonstrate ability; Administrative meetings (ex. SOT meetings)
	Interpersonal skills	Challenge not only self but others; Balancing technical with the relational; Negotiating with management and NGBs; Utilising network to their benefit and growth; Collaborating with HP teams within their organisation
	Self	Putting self forward; Having a clear vision; Identifying strengths

The most frequently mentioned ambition or goal by the participants pertained to their job role; 14 of the 17 coaches highlighted a desire to “move up” from their current job role or even move to more senior leadership roles outside coaching. For example, C2 expressed, “I want to be a high-performance coach… I'd like to be part of the team for Paris Olympics”. Another coach highlighted that in their current position they do not feel stretched enough and would wish to be able to coach either males or females at a much higher performance level –an opportunity they would relish (e.g., C17). Participants were interested in roles such as high-performance coach, head coach, performance director, national coach, senior/podium level coach, international/podium level coach. These roles were mentioned by the majority of the participating coaches. For example, one of the coaches said, “Performance Director is the ultimate role.” (C5). Four (C1, C6, C7, and C9) of the participants said they wished to stay in their current role for now, with C9 expressing ambiguity about her future role by mentioning the possibility of staying in her current role, moving sports, or even moving away from sport coaching to a lifestyle role. Nine of the participants expressed an immediate ambition to be able to attend or participate as a coach in major games or competition events, such as the Olympics, Paralympics, Commonwealth Games, European Championships, and World Championships at either the junior or senior levels. For example, “I definitely want to be involved with the senior team, with our Olympic team coaching.” C14. In addition, C5's desire to go to the Olympics is a key goal, “I've always said, I don't care how I go, but I'll go… So that's been me since about ten. You know, I'm going to the Olympics, I don't care how I'm doing it.”

The participants were also asked to discuss their plans for achieving their expressed goals or ambitions. Participants identified four focal areas that would help them achieve their goals and ambitions: knowledge, experience, leadership/interpersonal skills, and self (see Table 3). Gaining knowledge by attending courses, shadowing, increasing tactical/technical sport awareness in their respective sports, and learning about their aspired roles were mentioned by the participants. The value of a coach developer in achieving their professional career ambitions were also explained. Here is what C14 said about the importance of gaining knowledge of her aspired role, “Probably understand the set up within senior [name of sport] like the international set up, how the structure within our system actually works… really understand how things like selection works, how the pathway feeds into it; then within the actual senior set up internationally, how a cycle works.” And C16 expressed that “when we are allowed to travel, being able to go and look at athletes, coaches and athletes abroad” would aid her learning and knowledge growth which would ultimately facilitate her to reach her aspirations. C8 also mentioned utilising the networks she has developed to add to her experience, “work with certain key coaches in various countries that I already have links with”.

Experience and experiential learning were perceived as being instrumental in achieving what they desire. Some of the coaches were deeply involved in areas of their interest that while they were not directly coaching on the ground (e.g., coach development, collaborating with other coaches, driving strategical changes of the organisation) they saw as the necessary scaffolding for reaching their future professional goals. In addition, attending and importantly leading in major competitions, and opportunities to demonstrate the level of experience and competence gained were thought important, but such opportunities must be offered by the organisations. In demonstrating their ability, C16 added, “So if I can demonstrate that I've taken a cohort of athletes from their current level to Commonwealth Games and produce the medals that I predicted I could produce… I’m demonstrating my ability to be successful at the highest performance level of sport.” Coaches were certainly determined to advance, and one coach even said that she was prepared in order to gain the relevant experience to move sports, “And if I have to go to another sport to do it, I'll go to another sport to get opportunity, experience, and exposure at an appropriate level before coming back.” C5. Lastly, C15 mentioned gaining international experience by coaching for another country as an alternative plan for building reputation, experience, and skills.

Leadership and interpersonal skills, such as negotiating, networking, and relating as well as collaborating, making decisions and providing critical feedback were mentioned by the participants as key when they work with their athletes and their colleagues within and outside the organisation. One of the coaches mentioned the importance of finding the right balance between her relational (interpersonal) approach and her tactical or technical (competence) knowledge, “I work so much on the athlete relationship…I like listening and observing their body language and what they're saying and sometimes I think I go away from the technical. So it's like, how can I bring myself back to focusing on the technical and not being consumed by, feelings and personal stuff going on.” (C8)

Some of the participating coaches mentioned that the main plan of action for achieving one's ambitions is focusing on self. For example, one coach said, “putting [my] self forward… making my presence felt” C10 alongside having a clear vision, and identifying strengths are key aspects toward materialising one's goals. Another coach said, “just understanding what my strengths are or as a coach what are the strengths of my coaching that I am known for… And then, hone in on that and make sure that's something that nobody else ever has or it's something that I can really bring.” C14.

### Challenges

Two main areas were mentioned as being barriers or viewed as challenges in their trajectory as coaches: organisational and personal (see Table 4). Organisational barriers were regularly mentioned such as lack of funding or the volatility of a paid job and coaching courses to ensure personal development, culture of organisation, personal agendas, low number or lack of women coaches, stereotyping and biased perceptions from within organisation, to name a few. It was also felt that the coaching structure is set up to benefit coaches from overseas—usually white, male, impacting talented women coaches negatively. C1 expressed, “I feel like I’ve been marginalized” and “because the people that are in power at the moment, it's the people that employ coaches and it's their agenda.” C16 said she had to deal with people's false perceptions suggesting that her current coach role was due to her success as an athlete—“all last year I got a lot of that and I’m still getting some of it now.” Four participants felt that sport or their organisation needed to change, as C3 said, “I suppose a cultural shift that needs to happen within the organisation…we've never had a female coach working within any world class programmes”. C3, C10 and C12 spoke of the lack of women coaches within their sport, and C10 highlighted, “there's about four coaches. So at the moment, I'm one of those coaches and I'm the only female one.” and C12 expressed, “I think I can only name two female coaches actually, but we’re not seen in the field as well, which is really, really sad.” There was one coach who had not experienced stereotypes or biases though she admitted that this leadership development programme made her aware of how other women were challenged by stereotypes in coaching “while I never directly experienced it…it made me acknowledge that stereotypical bias exists” C17.

**Table 4:** Career challenges.

General category	1st order	2nd order
Challenges faced in career	Organisational	Lack of funding
		Lack of coaching courses
		Culture needs to change
		Personal Agendas
		Lack of other female coaches
		Coaches based overseas
		Stereotyping and perceptions
	Personal	Childcare needs
		Lack of confidence
		Language barrier

Personal challenges that were cited by participants were childcare needs and the pressure that this brings to individuals, a sense of lack of confidence or self-belief that is the result of the environment within which they worked, and a language barrier (that became evident when shadowing coaches in her sport that are experts, C3). When speaking about the dilemma created from her childcare needs and the schedule of training by her organisation, C2 said, “The relay practice is about two and a half hours, I've got children and there's no way, I've had to email them and say I can't do it… so they are going to say see, we gave you an opportunity…they are not looking at it from my perspective, I am a woman with children. And I'm a single parent as well.” C3 talked about her lack of confidence in soft skills, such as “communicating or negotiating… and asking for something a little bit more, it's these kinds of conversations I tend not to have.” She added that while she was confident in her technical and tactical skills, her lack of confidence in other areas might have caused her and her athletes to overlook important aspects of training within her organisation. However, she acknowledged that the programme made her aware of these potential shortcomings and that she is currently working to address them to prevent similar issues in the future.

### Recommendations

Several recommendations for the programme were made by all 17 participants and these were categorised in four main categories: theory and course content, mentor sessions, master coach sessions, and timing and logistics of the programme as well as continued support (see Table 5). Regarding the mentor sessions, participants suggested more such sessions and for them to be “longer” (C2) as well as Q&A sessions with invited high-profile coaches who operate in UK and abroad to share their journey and experiences. It was also felt that it would be of value to hear not only from other successful women coaches but from women who operate and have led successfully in other sectors, such as business. It was expressed that such content would not only build on their existing knowledge, but it would also provide them with inspiration and opportunities to model successful leaders.

**Table 5:** Recommendations.

General category	1st order	2nd order
Recommendations	Master coaches	Master coaches observe them in their environment and provide feedback
		Face-to-face meetings
		Cohort gets contact with all master coaches (Q&A panel, rotate coaches)
		Master coaches informed about what they are doing on the programme
		Master coaches share how they prep for major competitions (e.g., 6 months out from the Games)
		Harmonise pairings (Para coaches with Para master coaches, etc.)
		Having a theme or topic planned prior t the master coach meeting
	Theory and course content	Adding practical elements (role playing, public speaking opportunities)
		Building skills for difficult conversations (with athletes, how to deliver to PDs)
		Expand the topics for more interaction between the cohort (e.g., GROW model, difficult conversation)
		More time for discussions
		Cohort stretched more (held accountable for their learnings and how they applied them, etc.)
		More breakout groups
		Q&A sessions (accomplished women outside of sport, coaches of individual vs. team sport)
		Other topics including EI, teamship, problem solving, conflict resolution
		Instruction on how to apply the learnings to their athletes
	Mentor sessions	More and lengthier sessions
		Start programme earlier and extend its length
	Timing and logistics	Increase size of the small groups (more participants)
		Varying and mixing the groups
		Presentation at the beginning of the course about each participant and their journey
		Content more individualised (based around the journeys of each coach)
		Having a syllabus, more structured outline of the course and the objectives
		Support with building their network
	Continued support	Provide opportunities for furthering knowledge and skills and experience

Participants offered numerous recommendations to maximise the benefits of the master coaches work shadowing sessions including: (1) master coaches observing the participants in their own environment, (2) meeting with multiple master coaches with the aim to discuss pre-determined topics or themes (e.g., preparing from a major competition; work-life balance; negotiating a new role; leveraging relationships) and (3) while working with master coaches from different sports and types of sport (i.e., para and able bodied) was viewed useful, it was felt that pairing para-sport master coaches with para-sport coaches would equally be useful in terms of knowledge transference and expanding their insights. Participating coaches also suggested a list of topics that would be practically important for their coaching as this concerns their work on the ground. For example, effective communication (e.g., managing “difficult conversations with athletes” C4; “how we exit athletes off the programme” C14), leadership and relationships skills, emotional intelligence, the GROW model, team-ship, and problem solving. They also suggested more opportunities for group work and role-play scenarios. Related to this, it was also discussed that making learning as personalised or individualised as possible could make the development programme less like “it's like one size fits all.” (C6).

Concerning the timing and the logistics of the programme, participants provided recommendations such as starting the course “a bit earlier… if it starts in October, end of October, then it's quieter for a lot of people” C16 while lengthening its duration. It was also felt that increasing the size of the small group work because, “when we met, the reality was, there were only half of us there.” When participants were asked if they would like continued development support from UK Sport, all participants welcomed the notion (e.g., “I would 100 percent be [interested in further support]” C4; “I think it would be fantastic” C16. In addition, C2 said, “I think it's important that there has to be some accountability for the course that we’ve been on” and C2 also highlighted the benefit of tracking the cohort over time by capturing how the programme has made a difference to the participants, “Then you can say, well it's beneficial for these people because they've done this and they've done that, so why hasn't it work for those?”. Participants gave suggestions on how they would like to be supported along their journeys by UK Sport and two main areas were highlighted by the cohort, (a) support with building their network and (b) provide opportunities for furthering knowledge, skills, and experience.

The majority explained that forming a “community of practice” or a women coach network has benefited them throughout the program. They emphasised their desire to maintain, solidify, and expand it by organizing reunions with the cohort, possibly through monthly meetups. Additionally, they proposed hosting a yearly conference to celebrate women in coaching, facilitating connections with successful women both within and outside of sports, offering in-person shadowing opportunities, and continuing to use the WhatsApp group chat created for the program, alongside ongoing professional development. For example, C7 said, “I think the opportunity to have meet ups to discuss key topics and opportunities to have informal coffee chats, it would be massive.”

### Impact (results)

To capture the degree to which the programme was successful in terms of bringing about personal change to the participating coaches, we contacted all 20 coaches nearly three years following the completion of the programme. Responses via email were received by 10 coaches indicating that eight coaches were promoted since the completion of the programme and while one of the coaches was promoted to a leadership role within the same organisation, this leadership role was no longer a coach role. One of the coaches who reported no promotion explained that they had applied at least once for a more senior coach role to no avail. From these 10 coaches, four were selected to attend the Olympic (*n* = 1) and Paralympic (*n* = 3) Games in Paris as coach-related staff representing different sports.

## Discussion

A successful leadership programme is more than the sum of its parts and so the *Female Coaches and High-Performance Leadership Programme* was designed on three principles: (a) situate topics (i.e., vision, negotiation, networking) in an analysis of second-generation gender bias; (b) create a safe environment to support women coaches’ identity work; and (c) anchor women coaches on their leadership purpose while building important leadership skills for self-advancement. These principles were grounded on empirical evidence of what works especially for women who aspire to become leaders within their respective organisations (26, 48). Overall, the programme aimed to provide an understanding of how gender dynamics affect identity development in the workplace and a platform to support talented and ambitious women coaches to develop a leader identity.

Employing Kirkpatrick's (1998) four-level method (reactions, learnings, applications, results), we found that participating coaches had positive reactions about the leadership development programme. They positively evaluated the three interwoven strands of theory, small group mentoring sessions and master coaches’ work shadowing opportunities. It was evident that participating coaches had multiple opportunities to “*think themselves*” into a leadership role (49) while acknowledging the challenges of the broader environment within which they operated. COVID-19 restricted in person meetings to deliver the theory and the small groups mentoring sessions. Half of the participating coaches were unable to experience their master coaches in their work environments due to preparations for the Para/Olympics in Tokyo (2021). Alternative arrangements were made for these participants to meet with the master coaches online, somewhat curtailing the benefits of this offer. Nonetheless, for many the hybrid mode of delivery was viewed as flexible and suitable. Both, work shadowing and mentoring meetings allowed coaches space to “pause and think” about one's own career in high performance sport coaching. Many participating coaches appreciated the role of the coach developer in supporting them to become a better coach/leader and achieve their ambitions for the first time. The added value of coach developers aligns with recent research [e.g., (50, 51, 52)]. Generally, the results speak to the many ways the programme provided a window for both exploration and discovery of the participating coaches’ own sense of agency and purpose within a safe peer environment that was both extensive and diverse. In fact, efforts to maintain this group as a collective continue to-date with follow-up activities collaboratively organised by both UK Sport and UK Coaching. The benefits of such professional peer networks are immense as they can support women to initiate change for themselves and their organisations (23, 26).

There are sceptics that women-only programmes focus on “fixing women” [e.g., (18), p. 129] rather than dealing with organisational structures and cultures, known as the “uneven playing field” [(53), p. 11] inhibiting women to progress. While a couple of female coaches in the cohort expressed this sentiment, most of the participating coaches appreciated the difference a women-only leadership programme made to their development (20–22). First, the safe, non-judgemental environment the participating coaches created, comprised a strong network of like-minded people which may not have been the case in a mixed-sex development programme [see also (32)]. This safe place provided a platform to talk candidly about gender bias both overt gender bias that several had experienced and covert, subtle, almost invisible second-generation gender bias that some others experienced yet they were either less clear, unable to explain, or indeed not fully aware of it and the effects it had on them [see also (54)]. Second, participating coaches explained that they were able to see themselves as role models for the women in their sport (e.g., they felt energised to inspire and support other women) and trailblazers breaking down gendered career paths with a desire to coach male athletes and male teams, and gendered work whereby becoming a head coach was within their grasp. It became evident that these women coaches embraced the idea that “I am the change” and “We [women] are the change” and although the “system” (organisational structures and cultures) needs fixing, every single individual within the system has the agency to make a positive difference in diversity and inclusion and as such every person must be held accountable. This set of findings is consistent with research conducted in sport (4, 12, 55) and other fields (22, 56) suggesting the subtle challenges that women still must negotiate with resolve in the workplace.

Moreover, a women-only leadership programme is a novel context for many women coaches and such novel contexts have the capacity to shed light on more habitual or recurring domains [see (57, 58)]. Such programmes foster learning by putting women coaches in a majority position, provoking powerful insights. All the coaches felt they were in a powerful position to self-reflect and introspect, to consider their own career development and in some ways, they paused coaching and un-paused the coach/oneself. In this programme a gender-sensitive, critical eye purposefully not only opened women coaches’ horizons but also the horizons of organisations within which these women coaches operated in by reflecting on old-fashioned ideologies, policies, and practices. It revealed that women coaches and coaching as a profession still faces significant challenges: organisational [e.g., biases, stereotypes, remuneration, opportunities, recognition, funding (4, 9);] and personal [e.g., childcare, work-life balance, responsibility; (59, 60)]. The identification of such persisting challenges whereby at least some of them affect coaches regardless of their gender, should urgently drive national sport organisations to consider the “duty to care” that they must demonstrate for the coaches as they do for their athletes (61, 62).

The sponsors (individuals who put forward these women for a place in the programme) provided indirect feedback about the impact of the programme not only on the women coaches but also on them personally and the national sport organisations they represented (first' author's personal communication with various sponsors in the celebratory and concluding event of the programme in June 2021). The feedback received demonstrated that what is good for the women coaches can also be good for their sport organisations. In this study, sponsors can be instrumental to the progress, visibility, and opportunities women coaches experience. Lack of sponsorship may be what holds women coaches back from promotions and leadership positions. Sponsorship is a kind of helping relationship with people (sponsors) with power to advocate for, support, connect and place developing individuals in roles and assignments that expand their skillset and experiences and prepare them for senior positions are thought to be key [e.g., (56)]. Our work suggests that sponsors and potentially sponsorship programmes within national sport organisations is an important medium for unlocking high-potential women coaches’ careers, as well as increasing and retaining women coaches. These points indicate that more research is warranted.

Over the recent years, numerous women-only leadership and coaching development programmes have been designed and delivered (e.g., UK Coaching, UEFA, National Sport Organisations). These programmes have a place and some of them have reported an increase in the number and quality of women coaches actively pursuing a career in coaching (e.g., UEFA). However, it is important to note that coach/ing and leadership development can and should occur in a variety of venues utilising different methods and approaches over the course of coaches’ careers. Successful coaches learn from experiencing diverse environments that can be found both inside their sport organisations and outside them. Consequently, women coaches will do well to attend both women-only and mixed-sex programmes to achieve different objectives. Approximately a third of the coaches of this women-only programme subsequently gained places in a mixed-gender UK Sport-led coach programme.

The long-term impact of the programme provided some evidence of positive impact and successful results. Nonetheless, capturing the effectiveness of an educational programme like this one with precision would require researchers to track coaches’ professional journey even before the commencement of an educational programme, including turning points in their career and transitions into different and/or more senior roles post-programme. Practically, what we learned from the evaluation of this coach development programme can be summarised in the following four main points: (1) coach developers can be change makers on coaches’ professional journeys; (2) professional peer networking encourages conversation and collective thinking, broadens insights and can offer career opportunities—and it does not have to be all-women; (3) sponsors and by extension sponsorship programmes can be instrumental as they have the power to offer opportunities such as “stepping-stone jobs” to women coaches that lead to head coach positions; and (4) well-curated women only programmes offer a great deal of value especially when they are supported by a sport organisation/s that has a clear purpose and objectives for their existence. Practitioners (e.g., coach developers) and stakeholders (e.g., chief executive officers) across the world acknowledge that the demographics of the coach workforce need close attention and concerted effort if significant and positive change is to take place; we supplied here some practical ideas to support the change that is so desperately needed.

Overall, the UK Sport's Female Coaches Leadership Development inaugural programme for women coaches was experienced by the women coaches positively. Its efficacy was captured in terms of what was learned, what they were able to apply to their workplace and in pursuing and achieving promotions within their sport organisations. The programme shed a light on gender and identity-building offering them a better appreciation of the dynamics of gender in their development as a coach leader within their organisations. Moreover, these coaches were encouraged to clarify their mission and connect to a purpose larger than themselves. They continue to acknowledge, even to date, that the leadership development programme allowed room to think of their career as a coach and build their working identity. Subsequently, armoured with new knowledge, skills, and experiences, as well as renewed ambition, many of these women coaches were better prepared to take up and step into more senior coach leadership or other leadership-related roles in sport.

## Data Availability

The raw data supporting the conclusions of this article will be made available by the authors, without undue reservation.

## Footnotes

^1^The process for selecting the participants into the UK Sport's Female Coaches and High-Performance leadership programme was as follows. National Sport Organisations were contacted and invited to nominate prospective female coaches. The nominated participants were asked to apply directly to UK Sport. The criteria for identifying and nominating female coaches included: (a) to have approximately 10 years of coaching-related experience and (b) prospect to coach at major championships (Olympic and Paralympic Games) over the next 4 or so years—if they have not done already.
